# Modeling duration of overtaking between non-motorized vehicles: A nonparametric survival analysis based approach

**DOI:** 10.1371/journal.pone.0244883

**Published:** 2021-01-29

**Authors:** Yan Liu, Chuanyun Fu, Wei Wang

**Affiliations:** 1 School of Transportation, Southeast University, Nanjing, China; 2 School of Transportation and Logistics, Southwest Jiaotong University, Chengdu, China; Tongii University, CHINA

## Abstract

The use of non-motorized vehicles in urban city has improved the convenience of short-distance travel and reduced traffic pollution. However, the overtaking behaviour of non-motorized vehicles impacts traffic safety and efficiency significantly. The objective of this study is to model the durations of overtaking behaviour in the non-motorized vehicle exclusive lane. A total of 3010 overtaking events of non-motorized vehicles were extracted from two locations in Chengdu, China. The nonparametric survival analysis was conducted to model the overtaking duration of non-motorized vehicles. The categorical variables that significantly influence the overtaking duration were examined by the Log-rank test. The results show that the overtaking durations of female riders is longer than that of male riders. It takes longer for electrical bikes to complete overtaking than conventional bikes. When the non-motorized vehicle is under the load state (i.e. passengers or goods on the non-motorized vehicle), the overtaking behaviour takes more time than the un-load state. Moreover, it takes less time to overtake the non-motorized vehicle with load than to overtake the one without load. When there is a wrong-way driving phenomenon or under higher traffic volume, the duration is longer compared to the normal traffic and lower traffic volume conditions. The findings of this study attempt to provide a more profound understanding of non-motorized vehicles overtaking behaviour under different traffic conditions and give insights to the safety research of non-motorized vehicles.

## 1. Introduction

Non-motorized vehicles (NMV), including conventional bikes and e-bikes, have been an important component of the traffic system in many countries. Traveling with NMV can benefit the city environment, social economy, and public health [1, 2]. Many cities have developed exclusive lanes for the increasing demand of non-motorized traffic as supporting facilities. The dedicated lane spatially separates the non-motorized vehicles and the motorized vehicles, providing a relatively safe space for the non-motorized vehicles. As the decreasing volume of NMV, the interactions between non-motorized vehicles in their exclusive lane become a major concern affecting the NMV driving efficiency and safety. The overtaking behavior between non-motorized vehicles is a frequently observed interaction and potentially impacts the normal driving of NMV [3].

The overtaking event stems from the subject non-motorized vehicle’s pursuit to maintain the desired speed during riding. The significant speed difference between the subject NMV and the front NMV gives the possibility to complete the total overtaking event [4]. The overtaking behaviour is a complex physical and mental process that involves a series of actions including lane-changing maneuvers, acceleration phase, and deceleration actions [5]. Non-motorized vehicle riders have to evaluate the lateral space with the target NMV and adjust the speed and the riding angle. Increasing mental workload and pressure may have arisen during the judgments of the traffic environment and driving operations. The frequent changings in driving operations and cognitive pressure would deteriorate the driving safety and traffic efficiency. The NMV overtaking behaviour is a potential factor that increases the collision probability and downgrades the service of level. For example, the overtaking non-motorized vehicle can collide with another non-motorized vehicle or the curb due to false estimations of the lateral passing distance. The head-on may occur between the overtaking non-motorized vehicle and the wrong-way-driving NMV. Overtaking NMV may rear end with the front NMV owing to the abrupt change of the speed of the front NMV [6]. In addition, under non-physical isolation form and limited space driving space, the non-motorized vehicles invade to the motorized vehicle lane to pass the front NMV, which causes disorderly traffic conditions and NMV-MV accident [7].

Efforts have been made to analyze the characteristics of non-motorized vehicles during the overtaking maneuvers and the factors influencing the overtaking maneuver. The majority of studies focus on the speeds of the overtaking and overtaken bicycles, comfort speed difference threshold, lateral gap during passing, and the overtaking motivation. Khan et al. [8] examined the speed of bicycles during passing every 0.5 s through video recording and studied the relationship between the speed of the overtaking bicycle and that of the overtaken bicycle. This research also compared the lateral distance between the bicycle and the edge of the bicycle path during passing and no passing. Barmpounakis et al. [9] found that the speed difference between the overtaking vehicle and the front vehicle is the key factor that influences the powered-two-wheelers (PTW) overtaking maneuver. The observation research revealed several factors that affect the overtaking behaviour, such as whether there are passengers on the PTW, whether there are heavy vehicles in the road, and whether there is a platoon phenomenon. Guo et al. [10] investigated the lateral distance acceptance of the two-wheelers during overtaking. Findings show that the accepted lateral distance for bicycles is significantly larger than that for e-bikes and e-scooters. Zhao et al. [11] modeled the passing events in mixed bicycle traffic using cellular automata. It indicated that e-bicycles contribute to passing events in mixed traffic conditions. To the best of the authors’ knowledge, among the studies analyzing non-motorized vehicles’ overtaking maneuvers, no studies model the overtaking duration. However, the overtaking duration reflects the extreme hazard time that can be considered as a road safety evaluation parameter. As a matter of fact, the lane-changing maneuver and the acceleration and deceleration actions during the overtaking process make the road users more likely to generate traffic conflicts and violations. It is necessary to study the overtaking durations of NMV in order to improve the traffic safety level and better understand the traveling features of NMV. The further exploration of overtaking durations can also give inspires for the policy makers and designers about the non-motorized vehicle lane geometry designing, such as the lane width and the isolation form.

Although few studies have focused on the duration of overtaking behavior of NMV, the research on the duration of overtaking behaviour of motorized-vehicles is extensive, and the research methods can be used for reference. For instance, in order to model the duration of overtaking in two lane highways, Vlahogianni [12] applied the survival analysis approach to describe the total overtaking duration, accelerating phase duration, and the back-to-lane phase durations. Several exogenous variables were considered in the model as covariates to better understand the influence of different external conditions on the overtaking behaviour durations such as gender, speed, and available space. Another recent study analyzed the overtaking durations of motorcyclists [13], which is another type of two-wheeled vehicle, but larger in size and usually gasoline-powered compared to non-motorized vehicles, i.e. conventional bikes and electric bikes. A hazard-based duration model was developed to explore the difference of overtaking durations under different traffic conditions. The survival analysis, also called the duration model, is an appropriate method to model the duration data with a certain time as the starting time and a specific event as the ending point [14]. It has been frequently used in biology, medicine, and reliability engineering [15]. In recent years, survival models have also been widely applied in transportation researches, such as vehicles accident duration [16, 17], drivers’ reaction times for the traffic signal [18], riders’ waiting time of the crossing behaviour [19], drivers’ reaction times under mobile phone distraction [20], etc.

The present paper aims to model the durations of non-motorized vehicles’ overtaking behaviour in the non-motorized vehicle exclusive lane. Based on the field survey, the overtaking events were collected by video observations. The nonparametric survival model was carried out to analyze the overtaking durations of NMV. The difference of overtaking behaviour durations between male riders and female riders, different NMV types (bikes and e-bikes), and traffic environment were discussed using the Kaplan-Meier model.

## 2. Data collection

### 2.1. Study location

The map-based random sampling and field survey were used to select the study locations of this research. The following criteria were used for selecting the potential observation sites:

1. The type of isolation between the motorized vehicle lane and the non-motorized vehicle lane must be physical isolation. The non-motorized vehicle overtaking behaviour happen in both the physical isolated and non-physical isolated lane. However, overtaking events of non-motorized vehicles within the physical isolated exclusive lane can not be disturbed by the motorized vehicles, and it is much easier to observe the events and extract the durations.
2. The NMV volumes are large enough to allow sufficient overtaking events to be captured.
3. There is a pedestrian overpass at the upstream of the selected section for setting up the camera, and the road direction has a good view without obstructions.

Based on the abovementioned criteria, two sites in Chengdu, China, were selected based on Baidu Street View Map. They were the intersection of Dongxiu SecondRoad & Erhuan Road (site 1), and the intersection of Tongying Street & Erhuan Road (site 2). A pilot observation was conducted to ensure the reasonableness of the selection of the two survey sites. The two locations were visited on site to check the feasibility of video camera installation and the range of view. The traffic was also observed for 15 minutes to confirm the presence of overtaking behaviour between non-motorized vehicles.

### 2.2. Field observation

The non-motorized vehicle traffic of two intersection approaches were collected during January 2020. The weather condition was considered for video recording. Sunny days were chosen for a better field of view. The two study sites were recorded from 7:30 to 9:30 and from 12:00 to 14:00 on January 4^th^ and January 6^th^, 2020, respectively. The period of 7:30 to 9:30 was selected to represent the morning peak hours, and the 12:00 to 14:00 period was selected to represent the noon peak hours. Video cameras were installed in front of the approaches. The high-definition camera can capture the non-motorized vehicle flow state of the approaches for 2 hours at least, and support to extract overtaking events between NMV. A total of 8 hours of video data were collected at the two intersection approaches.

### 2.3. Extraction of overtaking behaviour between non-motorized vehicles

Video data were played using PotPlayer and observed by two analysts. To ensure inter-rater reliability, the two analysts were trained to understand the non-motorized vehicle overtaking behaviour, the duration of overtaking behaviour, and the factors that need to be recorded during observation. The two analysts were given a same short piece of video for practicing, and the extracted data were compared afterward. The principle is that the overtaking events and factors extracted by the two analysts are the same, and the average difference in the duration of overtaking behaviour is less than 5%.

**Fig 1:**
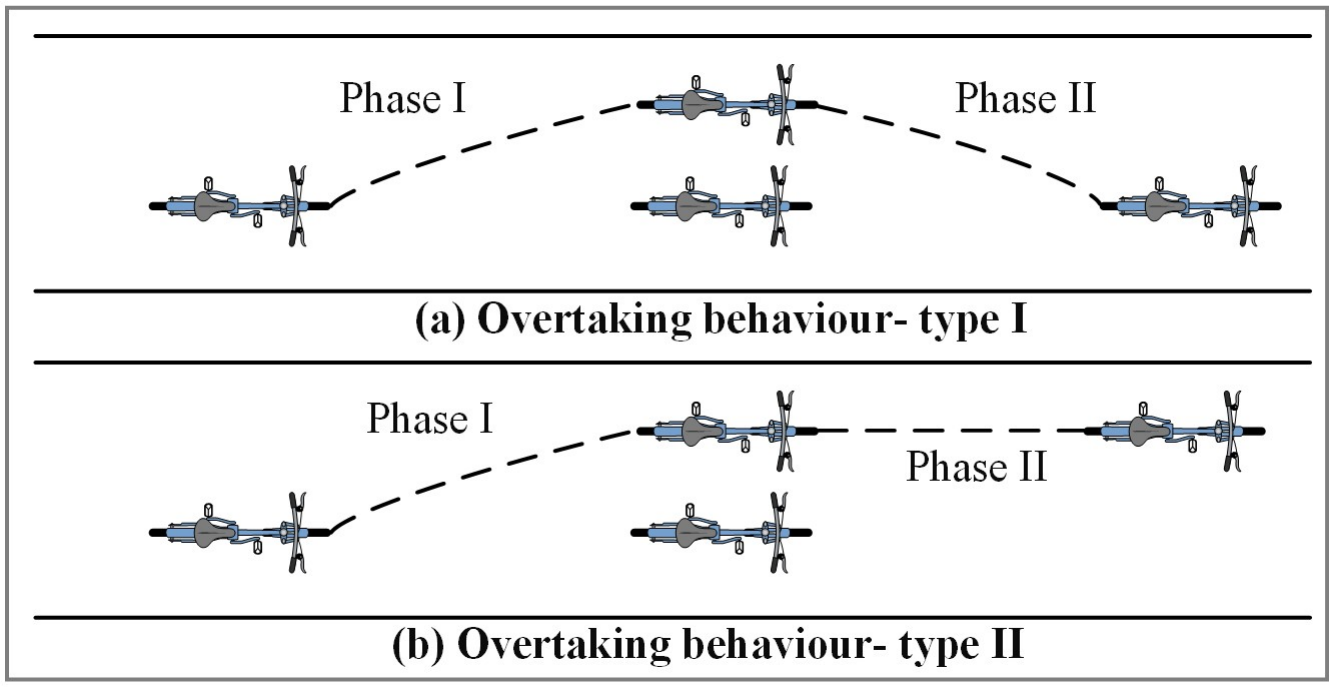
Graphical representation of the two types of overtaking behaviour.

In general, the overtaking behaviour can be divided into two phases, the acceleration-preparation phase (Phase I) and the overtaking-success phase (Phase II) (Fig 1). Phase I refers to the stage where the rider has the motivation to overtake and starts to change the steering angle and speed in order to overtake. In this phase, the rider begins to think whether to overtake or not and prepares to overtake the front non-motorized vehicle. Usually, the overtaking intention is the strongest when the speed of the front non-motorized vehicle is very low that hinders the riding of the rear non-motorized vehicle, and there is enough space for overtaking. Phases II refers to the after overtaking stage and the trajectory of the overtaking rider can be classified into two types. The first type is that the overtaking non-motorized vehicle turns back to the original lane position (Fig 1a). Contrarily, some riders choose to ride directly forward (Fig 1b). The choice of riding behaviour in the second phase depends on the riding habit of the rider, traveling space, and the surrounding traffic environment.

Based on the illustrations of the two types of overtaking behaviour, the starting and ending time of overtaking behaviour can be judged by the following principles. The starting time is the time when the non-motorized vehicle begins to change its trajectory in both of the two types of overtaking behaviour. The ending time of the two types of overtaking behaviour is different. For type I overtaking behaviour, the ending time refers to the time that the non-motorized vehicle overtakes successfully and back to the original lane position. For type II overtaking behaviour, the ending time is the moment that the rear of the non-motorized vehicle overtakes the head of the front non-motorized vehicle. The total duration of overtaking behaviour is the difference between the ending time and the starting time.

Potential factors considered to influence overtaking behaviour duration were also recorded. They include individual factors of the overtaking and the overtaken NMV, vehicle factors of the overtaking and the overtaken NMV, and traffic condition factors. The individual factors involve gender, phone use condition, and whether they wear helmets. The vehicle factors refer to the type and load status of non-motorized vehicles. Specifically, the non-motorized vehicle type includes bike and e-bike, and the load status refers to whether they carry passengers or goods. Traffic condition factors related to the riding environment include the traffic volume, whether there is on-street parking, whether there is pedestrian passing, and whether there are wrong-way driving (WWD) non-motorized vehicles. Typically, the average traffic volume per signal cycle is used as the traffic volume and is divided into two levels which are low (0–25 bicycle/cycle) and high (>25 bicycles/cycle). The wrong-way driving means traveling in the wrong direction or traveling in the opposite direction along the street [21, 22]. Besides, the time of day was also considered during video data recording (i.e. morning peak and noon peak).

## 3. Methodology

In the survival analysis model, the variable of interest is the time of duration, representing the survival time until a terminal event happens [15]. The terminal event in this study refers to the ending of overtaking behaviour Let T be a non-negative variable representing the time of an overtaking event, and the survival function S(t) is defined as the probability that T is of a length longer or equal to a specified time t, which means the overtaking event lasts to time t at least. S(t) is given by:

S(t)=1−F(t)=Pr(T>t) (1)

where F(t) is the cumulative distribution function, Pr is the probability.

The Kaplan-Meier (KM) method is a typical and most frequently used non-parametric model for estimating the survival probability of lifetime data, and also known as “Product-Limit model” [23]. The KM model provides an analysis approach that discusses the effects of influence factors on the duration by different analytical groups. The survival function in the KM model is [14]:

$$S(t)=\prod_{t_{i}\leq t}\left(1-\frac{d_{i}}{n_{i}}\right)$$ (2)

where *t*_*i*_ is the duration of the observation point i (i = 1, 2, 3, …), *d*_*i*_ is the number of events that happened at the time *t*_*i*_, *n*_*i*_ is the individuals that known to have survived before *t*_*i*_ (censoring has not happened until *t*_*i*_).

In this study, all the overtaking behaviours were fully recorded from the beginning to the ending, which means there is no censoring data. Furthermore, the overtaking events can be treated as recurrent events, because they occurred several times during the observation periods and the survival time for each overtaking event was unique. The survivor function can also be calculated using the recurrence relation, and is given as follows [14]:

$$S(t_{j})=S(t_{j-1})\times \text{Pr}(T>t_{j}|T\geq t_{j})$$ (3)

$$S(t_{j\text{-1}})=\prod_{\text{i}=1}^{j-1}\text{Pr}(T>t_{i}|T\geq t_{i})$$ (4)

where *t*_*j*_ is the No. j overtaking duration after ordering all the overtaking durations from smallest to largest, Pr(*T* > *t*_*j*_|*T* ≥ *t*_*j*_) is the estimated probability when the duration of overtaking is larger than *t*_*j*_ at least, and i and j are the index for an overtaking event.

The Log-rank test is applied to compare the significant difference between two or more different analytical groups. The null hypothesis of the Log-rank test is that there is no difference in the survival probability of an event at any time point [24]. The Log-rank test is a non-parametric test, which makes no assumptions about the survival distributions. In essence, the Log-rank test compares the observed number of events in each analytical group with the expected number of events if the null hypotheses were true, i.e. the survival cures were identical. The statistic indicator of the Log-rank test is approximately distributed as a chi-square test statistic, and can be calculated as follows [14]:

$$L_{LRS}=\frac{{\left(O_{2}-E_{2}\right)}^{2}}{Var(O_{2}-E_{2})}$$ (5)

$$O_{2}-E_{2}=\sum_{j=1}^{N}\left(m_{2j}-e_{2j}\right)$$ (6)

where *L*_*LRS*_ is the Log-rank test statistic, *O*_2_ is the observed score for the second group, *E*_2_ is the expected score for the second group, *m*_2*j*_ is the observed number of overtaking events when time is *t*_*j*_ in the second group, *e*_2*j*_ is the expected number of overtaking events when time is *t*_*j*_ in the second group, N is the total number of overtaking events and *j* ∈ [1, *N*].

## 4. Results and discussions

### 4.1. Descriptive statistics

**Fig 2:**
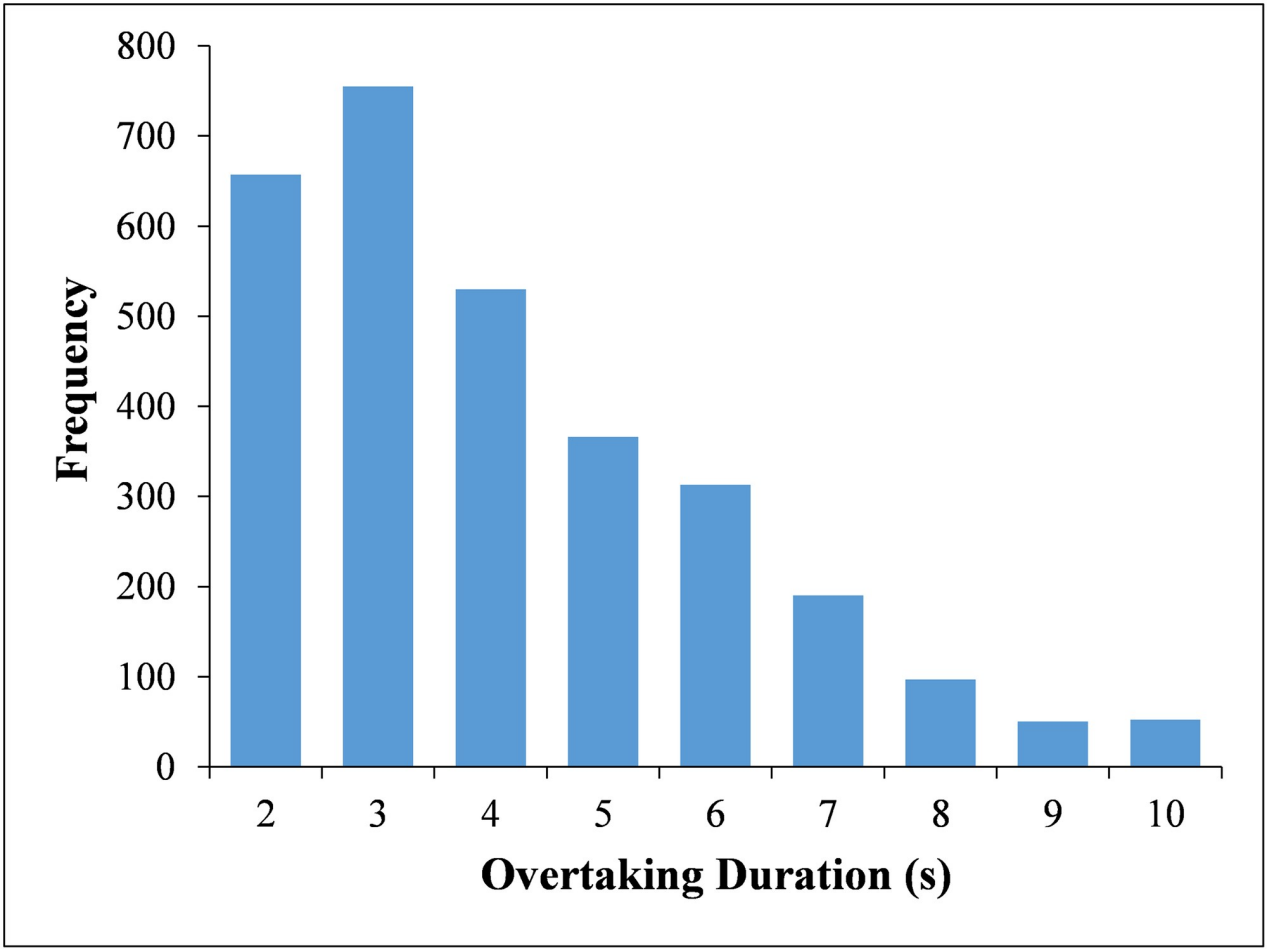
Histogram of overtaking duration.

From the 8 hours of video recordings, a total of 3010 valid samples were recorded. Table 1 depicts the distribution of the overtaking duration, and the histogram of overtaking duration is illustrated in Fig 2 for more visually display. The sample data of overtaking duration ranged from 1.153 s to 9.997 s, with a mean of 3.646 s and a standard deviation of 1.934 s. The observed 15th, 50th, and 85th percentile overtaking durations were 1.68 s, 3.15 s, and 5.80 s, respectively. A further look at the data shows that the majority of the observed overtaking durations are between 1 s and 4 s.

**Table 1:** Distribution of overtaking duration.

Overtaking duration (s)	Number of observations	Percentage (%)
1–2	657	21.83
2–3	755	25.08
3–4	530	17.61
4–5	366	12.16
5–6	313	10.40
6–7	190	6.31
7–8	97	3.22
8–9	50	1.66
9-Highest	52	1.73

### 4.2. Survival model estimation

Table 2 provides the results of the Log-rank test of different groups. Only the significant analytical groups at a 5% level of significance are included. The significant analytical groups are the gender of the overtaking rider, type of the overtaking NMV, load status of the overtaking NMV and the overtaken NMV, whether there is wrong-way driving, and the traffic volume level.

**Table 2:** Results of the Log-rank test.

Analytical groups	Log-rank test statistic	P-value
Gender of overtaking rider	260	< 0.001
Type of overtaking NMV	155	0.003
Load status of overtaking NMV	595	< 0.001
Load status of overtaken NMV	25.4	< 0.001
Wrong-way driving	234	< 0.001
Traffic volume level	10	0.002

**Fig 3:**
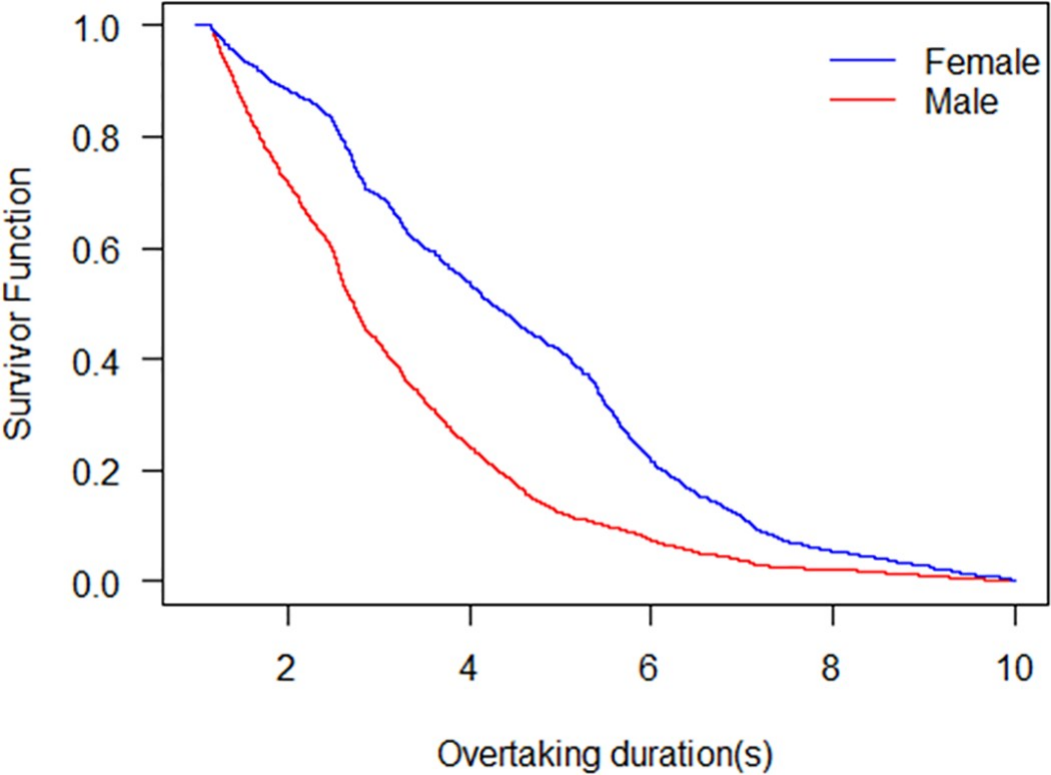
Survival probability of male and female overtaking riders.

The result of the Kaplan-Meier model indicates that longer durations of overtaking are anticipated for female riders than male riders on average (Fig 3). The survival curve means that the probability of failing to complete overtaking declines with the overtaking duration. The overtaking durations of male riders and female riders for median survival are 2.73 s and 4.25 s, respectively. For male riders, when the overtaking duration lasts 6 s, the overtaking behaviour finishes basically. However, when the duration is 6 s, the survivor function is still 0.2 for female riders. This result is in line with the fact that female riders are considered to be generally more prudent than male riders. An observation study indicated that females have a stronger sense of safety and drive slower than male drivers [25]. In general, the male riders are on average more experienced than female riders, and more predictable than female riders when overtaking [26]. Besides, female riders usually leave more space from the overtaken NMV than male riders [27], which makes female riders spend longer time to overtake than male riders.

**Fig 4:**
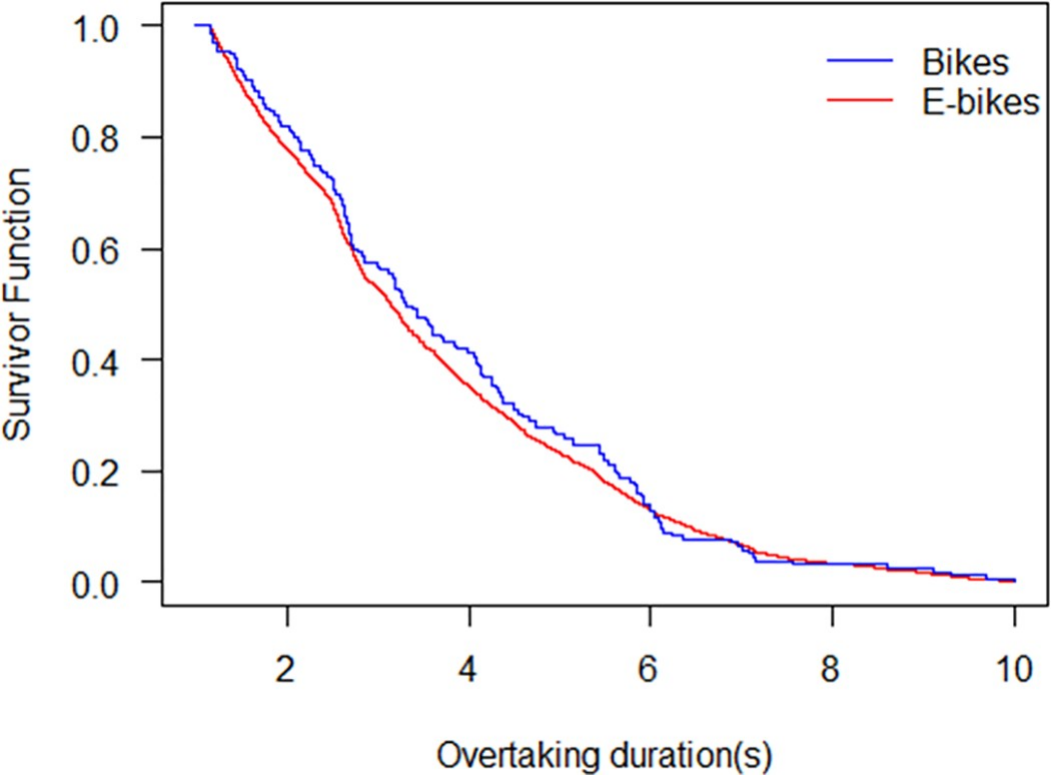
Survival probability of overtaking bikes and e-bikes.

The survival probabilities of overtaking durations for bikes and e-bikes are shown in Fig 4. The two survival curves are not far apart, which indicates that there is not much difference in the overtaking durations of bikes and e-bikes. It is observed that the ebikes and bikes ride in a comparable speed on the road. In general, the survival probability of bikes is a little higher than that of the e-bike at the same overtaking duration. One reason is that e-bikes are faster than bikes due to their different dynamical features [28]. A floating vehicle travel time study conducted in China has compared the speeds of bike and e-bike and shown that the speed of e-bikes is 30–35% higher than that of bikes [29], which results in a shorter time for e-bikes when they travel the same distance. Another reason is that the bike riders are more sensitive in the overtaking process than e-bike riders from the view of safety perception. A previous study has shown that the e-bike riders are riskier than conventional bike riders [30]. The survivor curves also show that after the duration lasts 6 s, the two survival curves almost coincide. This result is in accordance with the reality that the speed difference between bikes and e-bikes is not very significant at the observation sites. Specifically, the overtaking durations of bikes and e-bikes corresponding to the median survival probability for are 3.14 s and 3.31 s, respectively.

**Fig 5:**
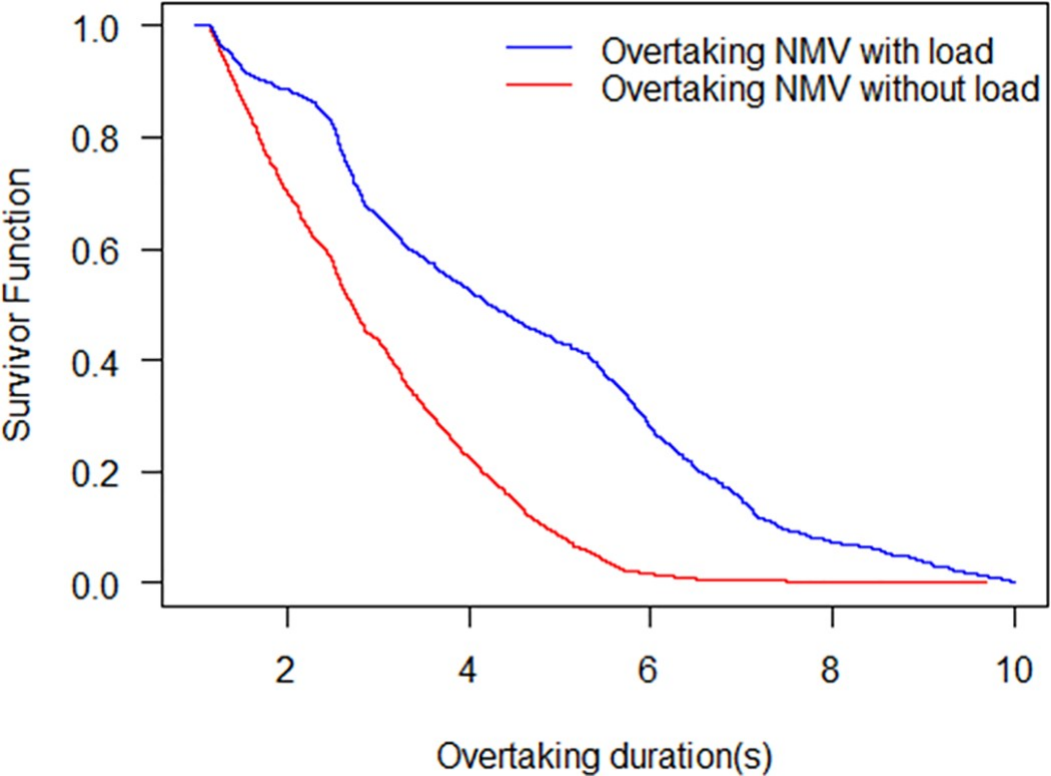
Survival probability of the overtaking NMV with load and without load.

With the Kaplan-Meier model, the survivor probabilities for overtaking NMV with load and without load are shown in Fig 5. The likelihood of failing to finish overtaking decreases with the overtaking duration. The survivor probability of the overtaking with load is higher than that of the overtaking without load status at the same overtaking duration. The results indicate that it takes more time for non-motorized vehicles to finish overtaking when they carry passengers or goods. The overtaking duration of the overtaking NMV without load for median survival is 2.72 s, while the corresponding value for the without load overtaking NMV is 4.22 s. For the overtaking NMV without load, the survivor function tends to be zero when the duration is 6 s. The KM analysis indicates that the survival function is close to zero when the whole overtaking duration is 10 s for the overtaking NMV with load. There are two reasons for the phenonmenon. First, it is observed that the overtaking NMV with load leaves a longer distance from the overtaken NMV during the overtaking period. Second, the speed of the overtaking NMV with load is lower than that without load.

**Fig 6:**
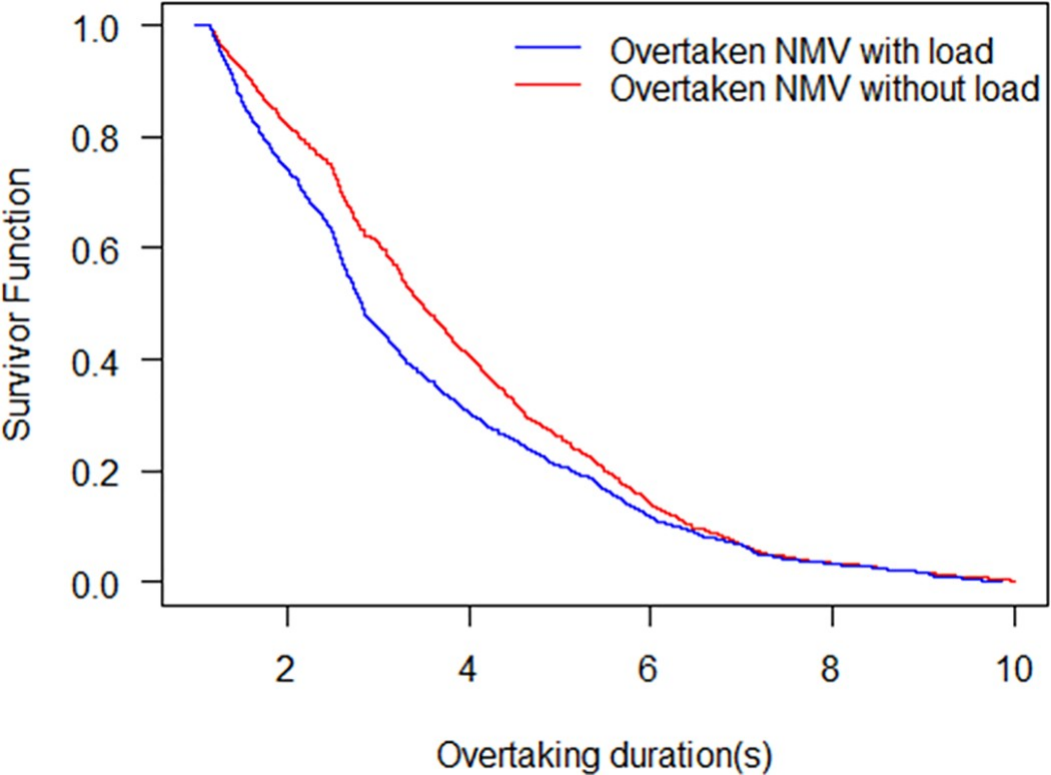
Survival probability of the overtaken NMV with load and without load.

The KM model estimation of the load status of the overtaken NMV is similar to the result of the load status of overtaking NNV. Fig 6 shows the survivor functions of overtaking durations when the overtaken NMV is with load and without load. The overtaking duration for median survival of the overtaken NMV with load is 2.83 s, while 3.48 s for that without load. It also indicates that after the overtaking duration lasts 6.2 s, the two survivor probability curves incline to coincide. In general, the riders of the overtaken NMV with load travel slower than the overtaken riders without load. From the video observation, the overtaken NMV with load occupied more road space than that without load, resulting in less space for overtaking than empty load state. In this circumstance, the overtaking riders need to slow down during passing, leading to a longer duration of overtaking.

**Fig 7:**
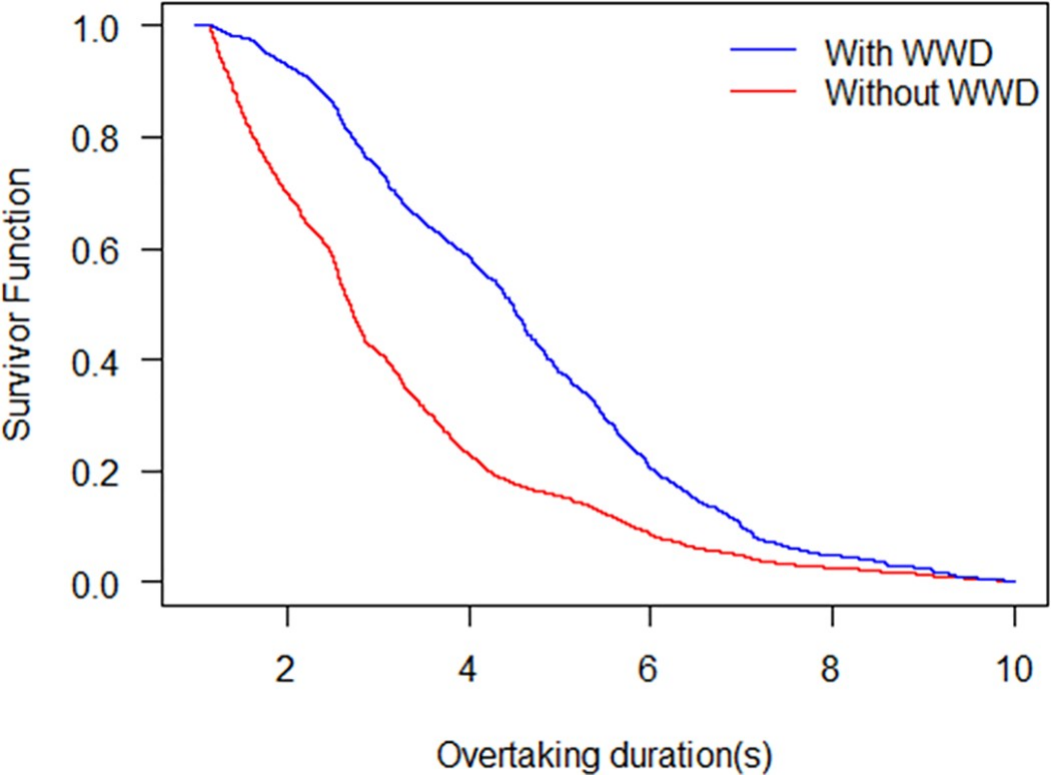
Survival probability with WWD and without WWD.

Fig 7 shows the survivor probabilities under the wrong-way driving condition and without wrong-way driving condition. The overtaking durations for median survival of the condition with WWD and without WWD are 2.70 s and 4.47 s, respectively. The result indicates that when there is WWD, the survivor probability is greater than that without WWD. It means that the wrong-way driving phenomenon leads to a longer overtaking duration. This case corresponds with the reality that wrong-way driving is a major constant traffic safety problem and reduces traffic efficiency. According to the previous study, WWD is the phenomenon that drivers travel in the opposite direction of traffic flow [31]. In the present paper, WWD refers to the condition that NMV riders travel against the main direction of the non-motorized traffic flow. In the situation of WWD exists, non-motorized vehicles in the main direction have to change speed and angle to avoid traffic conflicts with the WWD non-motorized vehicles. When the main driving non-motorized vehicle is in the overtaking process, overtaking duration will be increased by the wrong-way-driving non-motorized vehicles. The WWD phenomenon will lead to a reduction of traffic capacity and cause traffic congestion easily [32].

**Fig 8:**
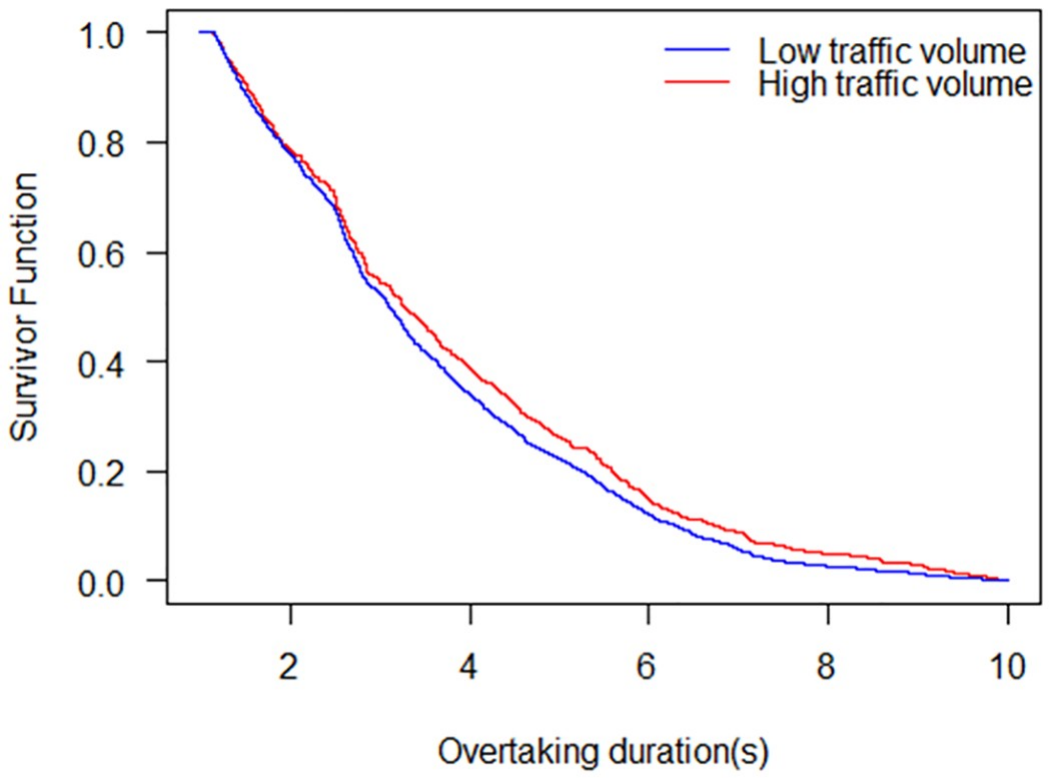
Survival probability under low traffic volume and high traffic volume.

The survivor functions of the durations of NMV overtaking behaviour under low traffic volume and high traffic volume are shown in Fig 8. The survivor probability under the low traffic volume is lower than that under the high traffic volume at the same overtaking duration. Specifically, the overtaking durations for the median survival under the high traffic volume and the low traffic volume are 3.27 s and 3.11 s, respectively. This result is in line with previous studies. A behavioural study found that the violation rate in high-density non-motorized vehicles condition was higher than that in low-density non-motorized vehicle conditions [33]. The increasing number of violations within the road will hinder the normal operations of traffic flow and affect the overtaking behaviour. This result is also consistent with the reality that speed decreases with the increase in traffic volume, which leads to a longer duration of overtaking behaviour.

## 5. Conclusions

This study applied a nonparametric survival analysis method to model the overtaking durations of non-motorized vehicles in the non-motorized vehicle exclusive lane. A total of 3010 non-motorized overtaking events were collected by video-based observations from two signalized intersection approaches in Chengdu, China. The mean value of duration conducted from the two observation sites is about 4 s, and most of the observed durations are between 1 s and 4 s. The relationship between overtaking durations and influencing factors are discussed in the Kaplan-Meier model. Results show that the overtaking durations of male riders are shorter than that of the female riders significantly. Different types of non-motorized vehicles have different overtaking time. Specifically, the duration of conventional bikes is longer than that of e-bikes. The load status of the overtaking non-motorized vehicle increases the duration, while the overtaken riders’ load status reduces the total duration. In addition, if there are wrong-way-driving vehicles, the duration will be enlarged. The overtaking duration is longer under high traffic volume than that under low traffic volume.

There are some shortcomings of this study. First, the data used in this study was from only two locations in China. The driving habits of NMV may vary among different traffic environments and countries. More observation sites and data set are recommended in future studies and the heterogeneity between observation sites can be considered in the model as well. Second, the overtaking durations analyzed in this study were extracted manually. The starting and ending points depend on the observers’ judgments. An emerging technology based on the computer-vision has been widely used in road safety analysis and evaluation [34–36]. Future work can use the trajectory tracking technology to get the road users trajectory information. The traveling parameters such as speed, acceleration, deceleration, and yaw rate can be considered in the model. The overtaking duration can also be considered in traffic conflict and traffic safety evaluation analysis in further studies with the help of automated video-based conflict analysis [37, 38]. Third, the influencing factors on overtaking duration are analyzed by analytical groups. The nonparametric model cannot quantify the effects of the variables. In the future study, the parametric models will be considered to better understand the influence factors.

## Supporting information

S1 Data (CSV)

## References

[1] Weinert JX, Ma C, Yang X, Cherry CR. Electric Two-Wheelers in China: Effect on Travel Behavior, Mode Shift, and User Safety Perceptions in a Medium-Sized City. Transp Res Rec. 2007; 2038(1): 62–68. 10.3141/2038-08

[2] Pucher J, Buehler R, Seinen M. Bicycling renaissance in North America? An update and re-appraisal of cycling trends and policies. Transp Res Part A Policy Pract. 2011; 45(6): 451–475. 10.1016/j.tra.2011.03.001

[3] Li Z, Wang W, Shan X, Jin P, Lu J, Yang C. Analysis of bicycle passing events for LOS evaluation on physically separated bicycle roadways in China. TRB 2010 Annual Meeting; Washington D.C.2010. p. 1–16.

[4] Jamson S, Chorlton K, Carsten O. Could Intelligent Speed Adaptation make overtaking unsafe? Accid Anal Prev. 2012; 48: 29–36. 10.1016/j.aap.2010.11.011 22664665

[5] Asaithambi G, Shravani G. Overtaking behaviour of vehicles on undivided roads in non-lane based mixed traffic conditions. J Traffic Transp Eng. 2017; 4(3): 252–261. 10.1016/j.jtte.2017.05.004

[6] Richter T, Ruhl S, Ortlepp J, Bakaba E. Prevention of Overtaking Accidents on Two-lane Rural Roads. Transp Res Proc. 2016; 14: 4140–4149. 10.1016/j.trpro.2016.05.385

[7] Liu Q, Sun J, Tian Y, Xiong L. Modeling and simulation of overtaking events by heterogeneous non-motorized vehicles on shared roadway segments. Simul Model Pract Theory. 2020; 103: 102072 10.1016/j.simpat.2020.102072

[8] Khan SI, Raksuntorn W. Characteristics of Passing and Meeting Maneuvers on Exclusive Bicycle Paths. Transp Res Rec. 2001; 1776(1): 220–228. 10.3141/1776-28

[9] Barmpounakis EN, Vlahogianni EI, Golias JC. Vision-based multivariate statistical modeling for powered two-wheelers maneuverability during overtaking in urban arterials. Transp Lett. 2016; 8(3): 167–176. 10.1179/1942787515Y.0000000020

[10] Guo Y, Sayed T, Zaki MH. Examining two-wheelers’ overtaking behavior and lateral distance choices at a shared roadway facility. J Transp Saf Secur. 2019: 1–21. 10.1080/19439962.2019.1571549

[11] Zhao D, Wang W, Li C, Li Z, Fu P, Hu X. Modeling of Passing Events in Mixed Bicycle Traffic with Cellular Automata. Transp Res Rec. 2013; 2387(1): 26–34. 10.3141/2387-04

[12] Vlahogianni EI. Modeling duration of overtaking in two lane highways. Transp Res Part F Traffic Psychol Behav. 2013; 20: 135–146. 10.1016/j.trf.2013.07.003

[13] Bella F, Gulisano F. A hazard-based model of the motorcyclists’ overtaking duration. Accid Anal Prev. 2020; 141: 105522 10.1016/j.aap.2020.105522 32283329

[14] David G. Kleinbaum MK. Survival Analysis: A Self-Learning Text. New York: Spinger; 2012.

[15] Washington SP, Karlaftis MG, Mannering FL. Statistical and Econometric Methods for Transportation Data Analysis, 2nd edition Boca Raton, Florida: Chapman & Hall/CRC; 2010.

[16] Chung Y, Walubita LF, Choi K. Modeling Accident Duration and Its Mitigation Strategies on South Korean Freeway Systems. Transp Res Rec. 2010; 2178(1): 49–57. 10.3141/2178-06

[17] Tavassoli Hojati A, Ferreira L, Washington S, Charles P. Hazard based models for freeway traffic incident duration. Accid Anal Prev. 2013; 52: 171–181. 10.1016/j.aap.2012.12.037 23333698

[18] Fu C, Zhang Y, Bie Y, Hu L. Comparative analysis of driver’s brake perception-reaction time at signalized intersections with and without countdown timer using parametric duration models. Accid Anal Prev. 2016; 95: 448–460. 10.1016/j.aap.2015.07.010 26211414

[19] Yang X, Huan M, Abdel-Aty M, Peng Y, Gao Z. A hazard-based duration model for analyzing crossing behavior of cyclists and electric bike riders at signalized intersections. Accid Anal Prev. 2015; 74: 33–41. 10.1016/j.aap.2014.10.014 25463942

[20] Haque MM, Washington S. A parametric duration model of the reaction times of drivers distracted by mobile phone conversations. Accid Anal Prev. 2014; 62: 42–53. 10.1016/j.aap.2013.09.010 24129320

[21] Pour-Rouholamin M, Zhou H. Investigating the risk factors associated with pedestrian injury severity in Illinois. J Safety Res. 2016; 57: 9–17. 10.1016/j.jsr.2016.03.004 27178074

[22] Fu C, Liu H. Investigating influence factors of traffic violations at signalized intersections using data gathered from traffic enforcement camera. PLoS One. 2020; 15(3): e0229653 10.1371/journal.pone.0229653 32130254 PMC7055877

[23] Kaplan EL, Meier P. Nonparametric Estimation from Incomplete Observations. J Amer Statistical Assoc. 1958; 53(282): 457–481. 10.1080/01621459.1958.10501452

[24] Bland JM, Altman DG. The logrank test. BMJ. 2004; 328(7447): 1073 10.1136/bmj.328.7447.1073 15117797 PMC403858

[25] Waylen AE, Mckenna FP. Is passenger presence associated with more or less risk taking? The 10th Seminar on Behavioural Research in Road Safety; London2001. p. 137–143.

[26] Walker I, Jones C. The Oxford and Cambridge Cycling Survey: A large-scale study of bicycle users in two major UK cycling cities. Oxford, UK: Oxford-shire County Council; 2005.

[27] Walker I. Drivers overtaking bicyclists: Objective data on the effects of riding position, helmet use, vehicle type and apparent gender. Accid Anal Prev. 2007; 39(2): 417–425. 10.1016/j.aap.2006.08.010 17064655

[28] Cherry C, Cervero R. Use characteristics and mode choice behavior of electric bike users in China. Transp Policy. 2007; 14(3): 247–257. 10.1016/j.tranpol.2007.02.005

[29] Cherry C. Implications of electric bicycle use in China: analysis of costs and benefits. Berkeley, CA: UC Berkeley Center for Future Urban Transport-Volvo Summer Workshop; 2006.

[30] Haustein S, Møller M. E-bike safety: Individual-level factors and incident characteristics. J Transp Health. 2016; 3(3): 386–394. 10.1016/j.jth.2016.07.001

[31] Baratian-Ghorghi F, Zhou H. Traffic control devices for deterring wrong-way driving: Historical evolution and current practice. J Traffic Transp Eng. 2017; 4(3): 280–289. 10.1016/j.jtte.2016.07.004

[32] Kuang X, Cao W, Wu Y. Cellular automata model of non-motor vehicle flow considering reverse vehicles. J Syst Simul. 2016; 28(2): 268–274.

[33] Zhang W, Zhou C, Huang W, Tao H, Wang K, Feng Z, et al Investigating factors affecting riders’ behaviors of occupying motorized vehicle lanes on urban streets. Accid Anal Prev. 2019; 122: 127–133. 10.1016/j.aap.2018.09.025 30343164

[34] Fu C, Sayed T, Zheng L. Multivariate Bayesian Hierarchical Modeling of the Non-Stationary Traffic Conflict Extremes for Crash Estimation. Analytic Methods in Accident Research. 2020: 100135 10.1016/j.amar.2020.100135

[35] Guo Y, Essa M, Sayed T, Haque MM, Washington S. A comparison between simulated and field-measured conflicts for safety assessment of signalized intersections in Australia. Transp Res Part C Emerg Technol. 2019; 101: 96–110. 10.1016/j.trc.2019.02.009

[36] Guo Y, Sayed T, Zheng L, Essa M. An extreme value theory based approach for calibration of microsimulation models for safety analysis. Simul Model Pract Theory. 2021; 106: 102172 10.1016/j.simpat.2020.102172

[37] Guo Y, Sayed T, Zheng L. A hierarchical bayesian peak over threshold approach for conflict-based before-after safety evaluation of leading pedestrian intervals. Accid Anal Prev. 2020; 147: 105772 10.1016/j.aap.2020.105772 32949863

[38] Guo Y, Sayed T, Essa M. Real-time conflict-based Bayesian Tobit models for safety evaluation of signalized intersections. Accid Anal Prev. 2020; 144: 105660 10.1016/j.aap.2020.105660 32623321

